
JOURNAL INFORMATION
==============================
NLM Title Abbreviation: Anim Front
Journal ID: Anim Front
NLM Journal ID: 101754918
Journal ID: anfron

Animal Frontiers: The Review Magazine of Animal Agriculture

ISSN: 2160-6056
EISSN: 2160-6064
Publisher: Oxford University Press

ARTICLE INFORMATION
==============================
PMCID: PMC11377065
DOI: 10.1093/af/vfae021
Article ID: vfae021
Article version: 1
Subjects: Society News, AcademicSubjects/SCI00960

News

Publication date: 2024 Aug
Electronic publication date: 2024 Sep 5
Volume: 14
Issue: 4
First page: 32
Last page: 37
Copyright: Copyright © 2024 American Society of Animal Science
Copyright year: 2024
License: This is an Open Access article distributed under the terms of the Creative Commons Attribution License (https://creativecommons.org/licenses/by/4.0/), which permits unrestricted reuse, distribution, and reproduction in any medium, provided the original work is properly cited.
License URL: https://creativecommons.org/licenses/by/4.0/

Page count: 6

==============================

### WORLD ASSOCIATION for ANIMAL PRODUCTION

The World Federation of Animal Science Societies and Associations, **WAAP**, is an International Non-Governmental Organisation with special consultative status with FAO since 1974.
The WAAP was established in 1965 after the success of the 1st World Conference on Animal Production held in Rome in 1963 organised by EAAP under the patronage of FAO.
WAAP aims to encourage closer collaboration between the animal production organisations and to bring together scientists, educators, technicians and administrators with the objective of reviewing development concerns on the five continents.

The Melbourne city at dusk

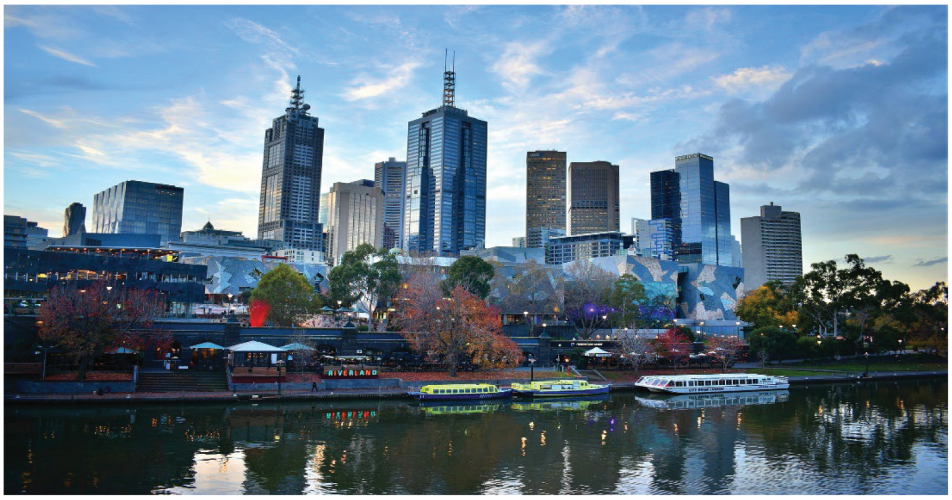

WAAP organises every five years a World Conference on Animal Production. Co-organized by EAAP and WAAP, the 2023 conference on “Climate change, biodiversity and global sustainability of animal production”, has been held in Lyon end August. It received 1900 abstracts among which 1200 were seleted for oral presentations in 96 sessions. More than 2200 delegates came to the conference. Ten sessions, organized or co-organized by WAAP, were video recorded and will be soon available by streaming on the EAAP Youtube channel. Two of them were especially attractive on the “Control of GHG emissions in ruminant farming” and on “Biodiversity as a lever for sustainable animal production”.

During the conference, WAAP delivered senior and junior awards and 3 student grants.

Philippe Chemineau ended his mandate after 5 years, Franck Dunshea was elected as the new President for 5 years. He will be in charge of organizing the next WCAP which will be held in Melbourne, Australia, in 2028.

https://doi.org/10.1093/af/vfae013

American
Meat
Science
Association

### *Meat and Muscle Biology*

**Premier, International, Gold Open Access journal**

For a broad scope of high quality, peer-reviewed manuscripts on all aspects of meat science, applied muscle biology, and related topics.

### *MMB* EDITORIAL BOARD

- TOMMY WHEELER, PH.D.
- LINDA SAUCIER, PH.D.
- CLETOS MAPIYE, PH.D.
- SURINDER SINGH CHAUHAN, PH.D.
- MASSIMILIANO PETRACCI, PH.D.
- HYUN-WOOK KIM, PH.D.
- DEQUAN ZHANG, PH.D.
- JERRAD LEGAKO, PH.D.
- TRAVIS O'QUINN, PH.D.
- MARIO CHIZZOTTI, PH.D.
- WES SCHILLING, PH.D.
- ALEX STELZLENI, PH.D.
- CHEORUM JO, PH.D.
- JEAN-FRANÇOIS HOCQUETTE, PH.D.
- ANDREA GARMYN, PH.D.
- JEFF W. SAVELL, PH.D.
- ARGENIS RODAS-GONZÁLES, PH.D.
- ELISABETH HUFF-LONERGAN, PH.D.
- RANJITH RAMANATHAN, PH.D.
- QUN SUN, PH.D.

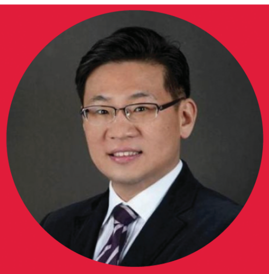

**BRAD KIM, PH.D.**
Editor In Chief
*Purdue University*

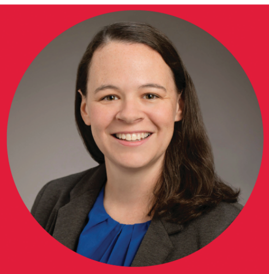

**ANNA DILGER, PH.D.**
Associate Editor
*University of Illinois*

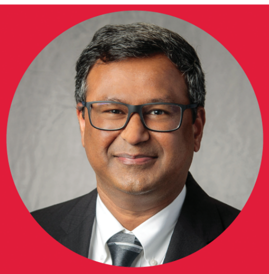

**SUMAN SURENDRANATH, PH.D.**
Associate Editor
*University of Kentucky*

All Species. All Disciplines. All the Time.

**Submit with MMB Today!**

For more information visit: **WWW.MEATANDMUSCLEBIOLOGY.COM**

AMERICAN SOCIETY OF **ANIMAL SCIENCE**

The "Biology of Lactation in Farm Animals" (BOLFA) and the International Congress on Farm Animal Endocrinology (ICFAE) meetings will be held at the University of Bern, Switzerland, as satellites of the annual EAAP meeting in Florence, Italy. The meetings in Bern take place from August 28-30, 2024. Current topics of lactation and endocrine systems in farm animal species will be discussed during the 3-day conference.

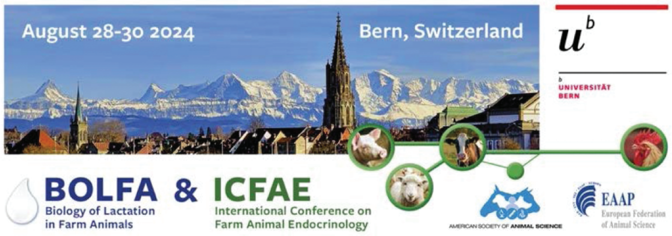

There is still time to register! You do not want to miss this comprehensive meeting.

The 2025 Midwest Section Meeting will take place in Omaha, Nebraska, March 9-12, 2025, at the CHI Health Convention Center.
Abstract submissions will open in September 2024. Check the meeting webpage, https://www.asas.org/sections/midwest-section/meetings, for updates.

AMERICAN SOCIETY OF **ANIMAL SCIENCE**
**MIDWEST SECTION**

Join us in Arlington, TX April 5-8, 2025, at the Sheraton Arlington Hotel for the joint meeting of ASAS Southern Section and ASAS Western Section. More information coming soon, https://www.asas.org/sections/southern-section/meetings.

SAVE THE DATE
ASAS-CSAS Annual Meeting, Hollywood, Florida, July 6-10, 2025.

As a member of ASAS, we are sure you are aware of all the benefits that having a professional membership offer, including complimentary registration to monthly webinars and early access to the recordings, access to ASAS Journals, and discounted meeting registration to name a few. Remember to renew your ASAS membership or join ASAS to take advantage of these benefits.

More information on memberships can be found on the Membership website, https://www.asas.org/join-asas/individual-membership.

EAAP
European Federation of Animal Science

**THE 75$^{th}$ EAAP ANNUAL MEETING WILL BE HELD IN FLORENCE (ITALY) FROM 1$^{st}$ TO 5$^{th}$ SEPTEMBER 2024**

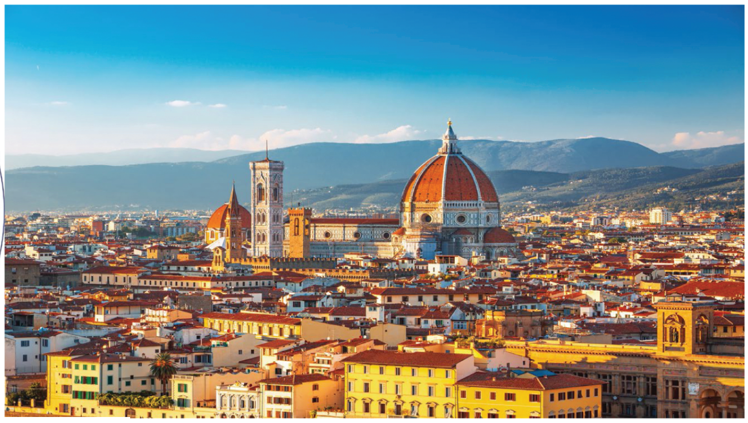

On behalf of the Italian Organising Committee, we are very delighted to invite you to attend the 75$^{th}$ EAAP Annual Meeting 2024 to be held in Florence (Italy), from 1$^{st}$ to 5$^{th}$ September, 2024 at Firenze Fiera congress Center.
The general topic of the Congress will be "**Global quality: environment, animals, food**".
The sessions will be organized and jointly organized between the eleven EAAP Study Commissions on genetics, nutrition, physiology, animal health and welfare, precision livestock farming, horses, pigs, insects, livestock farming systems, cattle, sheep and goat.
We received an incredibly high number of abstract submissions, a record for the Annual conference only: more than 1800 abstracts compared to about 1300 in previous annual EAAP meetings. These abstracts come from 60 different countries from all over the world!
The title of the Plenary session that will be organised on September 2$^{nd}$ August is:
**Reassessing our Relationship with Farmed Animals.**
Italy owns the largest number of PDO and PGI products.
Different technical tours will be organised to discover the Mediterranean Livestock Faming systems and Biodiversity (Beef cattle - Maremmana, Cinta Senese pigs, Dairy buffalo farming), the Tuscany agricultural landscapes (Chianti hills, rural mansions, Wineries), the high-quality DOP productions of Parmigiano Reggiano, Prosciutto di Parma and San Daniele, Pecorino Toscano and Prosciutto Toscano.
We welcome you and wish you an exceptional stay in Florence!

**Congress Website:**
http:// www.eaap2024.org
**General information:**
eaap2024@thetriumph.com
**Sponsorship and exhibition opportunities:**
sponsoreaap2024@thetriumph.com

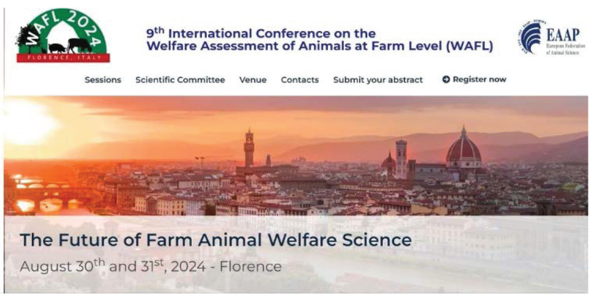

The **9th International Conference on the Welfare Assessment of Animals at Farm Level (WAFL)** will be held on August 30th and 31st, 2024 in Florence
Early Bird registration deadline is extended to **June 15th**!
Don't miss the opportunity to participate to this special event taking advantage of the discounted price offered by the Early Bird registration!
**Congress Website:** https://wafl2024.eaap.org/#home

Please follow us on Facebook and Twitter
https://www.facebook.com/EAAP.ORG
https://twitter.com/eaapofficial
https://www.linkedin.com/company/european-federation-animal-science
Instagram page: eaap_official

INDIVIDUAL MEMBERSHIP OF EAAP

The EAAP membership is open to all scientists. It is a great opportunity to be update on the latest publications and other relevant information in the animal sector.
We have more than **6000 members** already!
Become a member of the most exciting international animal science network, joining us at http://www.eaap.org/Content/Individual_Member_Information.html

THE EAAP NEWSLETTER FOR INDIVIDUAL MEMBERS IS NOW AVAILABLE ALSO IN CROATIAN, FRENCH, GERMAN, PORTUGUESE, ROMANIAN, SLOVENIAN, SPANISH, SLOVAK, ITALIAN AND POLISH!
